# Recurrent Adolescent Giant-Cell Tumor of the Scaphoid: Scaphoid Excision with Intracarpal Fusion after Failed Curettage and Bone Grafting

**DOI:** 10.1155/2019/7571486

**Published:** 2019-04-09

**Authors:** Chris Hoedt, Gabriel S. Makar, Christina J. Gutowski, Thomas Holdbrook, Tae Won B. Kim, David A. Fuller

**Affiliations:** ^1^Cooper University Hospital, Department of Orthopaedic Surgery, 3 Cooper Plaza Suite 410, Camden, NJ 08103, USA; ^2^Cooper Medical School of Rowan University, 401 Broadway Ave, Camden, NJ 08103, USA; ^3^Cooper University Hospital, Department of Pathology, 1 Cooper Plaza, Camden, NJ 08103, USA

## Abstract

We present a case of the giant-cell tumor of bone in the scaphoid of a 17-year-old female. Imaging revealed an expansile lytic lesion of her scaphoid, and the diagnosis was confirmed with open biopsy. She was treated with curettage and iliac crest bone graft, in an effort to spare reconstruction of her wrist. After one year, she developed increasing tightness and pain. Local recurrence was apparent on radiographs, and CT revealed increased lucency with bony destruction in the area of prior excision. She was successfully treated, without recurrence to date, with complete scaphoid excision and a four-corner wrist fusion. Local recurrence of the giant-cell tumor of bone is high, especially in carpal bones. When treating patients with advanced lesions, more aggressive initial options should be considered.

## 1. Introduction

Hand tumors are relatively uncommon with 15% of soft tissue tumors and 6% of bone sarcomas occurring in the wrist and hand [1–4]. Tumors of the carpal bones are rare with a prevalence of 0.16% [5]. The giant-cell tumor of bone (GCTB) comprises approximately 2-5% of hand tumors with most occurring in the metacarpals and phalanges [6–8]. GCTB is a benign but locally aggressive osteolytic tumor characterized by the presence of multinucleated, osteoclastic giant cells. It accounts for approximately 5% of all primary bone tumors and occurs most often in the epiphyses of long bones in the third or fourth decade of life [9]. The Campanacci classification grades these tumors based on radiographic characteristics. Local recurrence rates are variable in the literature, ranging from 8 to 45%, depending on the extent of tumor removal, use of chemical/topical adjuvants, and other host factors [10–12]. Benign metastases develop in approximately 2% of patients [9]. Treatment is commonly extended intralesional curettage with adjuvant treatment. However, en bloc resection of these tumors has been shown to be associated with reduced recurrence rates and may be indicated for carpal lesions that are grade 2 or 3 lesions or recurrent tumors.

Risk factors for local recurrence include patient age at diagnosis, Campanacci grade, use and type of adjuvant therapy, use of medical therapy, primary versus recurrent tumor, anatomic location, and packing technique [10–12]. Tight and complicated anatomy of the hand and foot may complicate adequate extended curettage and adjuvant treatment. Rajani et al. reviewed 18 patients with giant-cell tumors of the foot and ankle bones: of 17 tumors treated with intralesional procedures, 10 developed recurrence [13]. Local recurrence in the hand is even more prevalent, with reported rates of up to 90% following curettage and bone grafting [8, 14]. Only 2% of all GCTs have been reported in the hand, with 10% of these arising in the carpal bones [8, 14]. To date, only six GCTs of the scaphoid have been reported in the literature, with no known cases under the age of 18 [15].

The patient we report was informed that data concerning her case would be submitted for publication, and she provided consent.

## 2. Case Report

**Figure 1:**
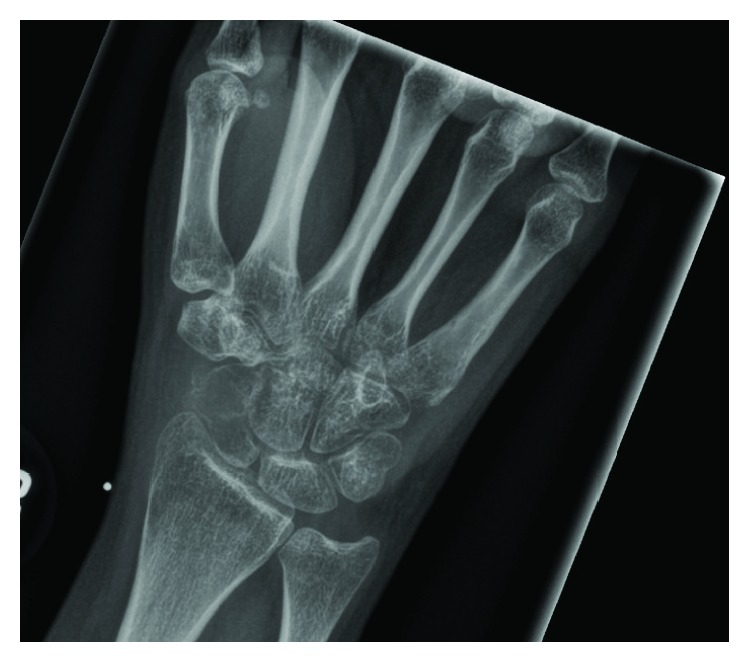
Diagnostic AP XR. AP radiograph of the right wrist, demonstrating radiolucency within the scaphoid.

**Figure 2:**
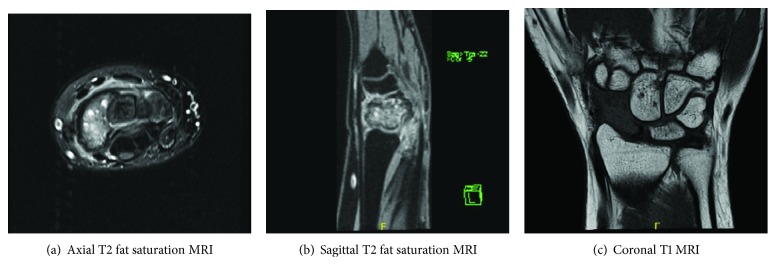
Axial T2 fat saturation (a), sagittal T2 fat saturation (b), and coronal T1 (c) MRI sequences of the right wrist, demonstrating a marrow-replacing bone lesion within the scaphoid.

A 17-year-old right-hand dominant female presented with atraumatic, progressive, activity-related right wrist pain for five months. Clinical examination showed tenderness over the scaphoid with a limited range of motion and decreased strength compared to her left wrist. Wrist radiographs revealed a lytic lesion of the scaphoid with a nondisplaced pathologic fracture (Figure 1), and MRI demonstrated a marrow-replacing expansile lesion with extraosseous extension and multiple fluid-fluid levels (Figures 2(a)–2(c)).

**Figure 3:**
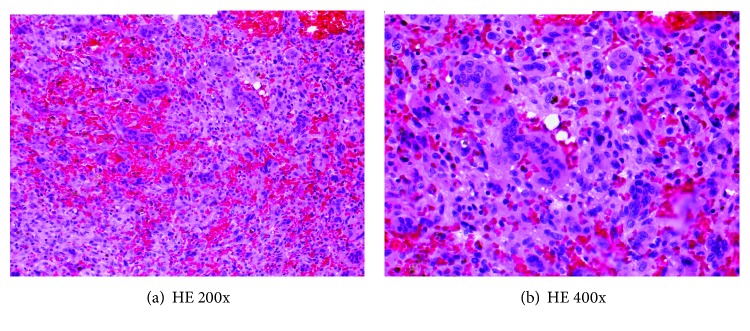
(a, b) Histopathologic examination demonstrating uniformly distributed multinucleated giant cells among mononuclear stromal cells; the nuclei of the giant cells and the stromal cells appear identical.

**Figure 4:**
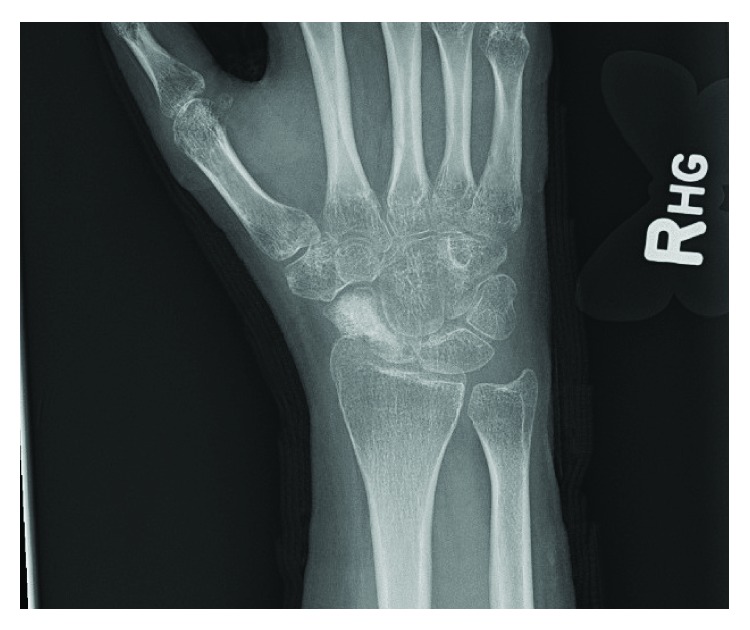
AP radiograph of the right wrist, demonstrating early consolidation of the autograft without evidence of local recurrence. 4-month follow-up X-ray.

An open biopsy from the volar approach and intraoperative frozen section revealed the giant-cell tumor of bone. A volar approach for the biopsy was selected to allow complete access to the scaphoid since the lesion was Campanacci grade 3 and a dorsal approach may have limited the operative area. The lesion was curetted, electrocautery was applied to the surfaces of the defect, and it was packed with iliac crest bone autograft including a corticocancellous strut; pathology confirmed the diagnosis (Figures 3(a) and 3(b)). The patient tolerated the procedure well. She wore a long-arm thumb spica cast for 12 weeks and used a bone stimulator from week 6 to 12. At her 4-month follow-up, she was transitioned to a splint and began occupational therapy, and her X-rays showed early consolidation of the graft without displacement or obvious local recurrence (Figure 4). At her 4-month exam, she had 25 degrees of wrist flexion and 25 degrees of extension. She had full motion and function of all of her fingers and could oppose all fingers to her thumb without difficulty.

**Figure 5:**
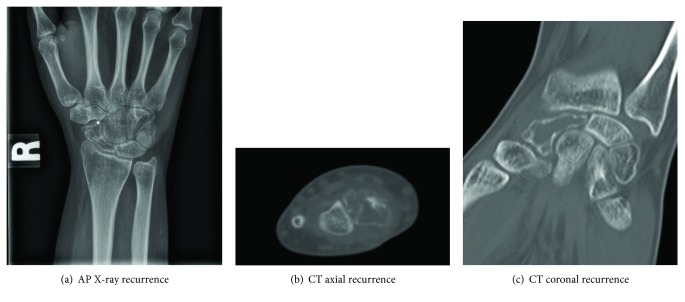
(a–c) AP radiograph (a) and axial and coronal CT scans (b, c) of the right wrist, demonstrating increased lucency and graft resorption within the scaphoid, consistent with local recurrence of the giant-cell tumor of bone.

At her 1-year follow-up visit, she complained of increased tightness and intermittent pain in her wrist. She had lost the ability to comfortably flex her wrist, but otherwise, her exam was unchanged. Her wrist X-rays showed an interval lucency within the scaphoid, and CT scan demonstrated cystic appearance within the scaphoid and demineralized cortical rim, concerning for tumor recurrence (Figures 5(a)–5(c)).

**Figure 6:**
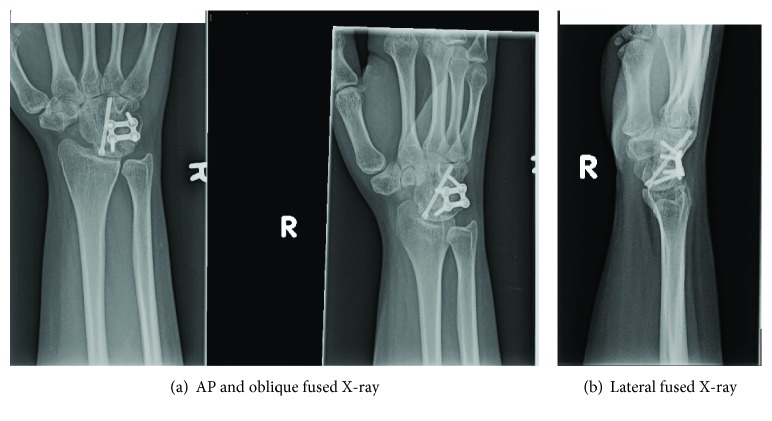
(a, b) AP and oblique (a) and lateral (6) radiographs of the right wrist, demonstrating consolidated 4-bone carpal fusion.

Fifteen months after her initial procedure, she underwent complete excision of her scaphoid and a four-corner wrist fusion. Intraoperative pathology assessment confirmed recurrence of the giant-cell tumor of bone. She was placed in a thumb spica splint for 6 weeks, then transitioned to a removable splint. At 12 weeks postoperative, she began occupational therapy. At her one-year follow-up from her excision and fusion, she was back to work and pain-free. She could extend her wrist 20 degrees and flex 15 degrees. She had 70 degrees of pronation and supination. She could radially deviate her wrist 5 degrees and ulnarly deviate her wrist 10 degrees. At her one-year follow-up, her grip strength was 5/5. Her follow-up X-rays taken at that visit showed a well-healed fusion of the lunate to the capitate to the hamate to the triquetrum (Figures 6(a) and 6(b)).

## 3. Discussion

Primary bone tumors of the hand and wrist are rare. Soft tissue tumors are more common than bone tumors with benign lesions being more common in both instances [16, 17]. Bone tumors of the hand and wrist comprise approximately 4-7% of all bone tumors, most of which (86%) are benign [18, 19]. Metastases to the hand comprise only slightly more than 0.1% of total bone metastases [20]. Overall, tumors of the hand and wrist are highly uncommon with primary bone tumors being especially rare.

We present the first adolescent case of the giant-cell tumor of bone arising in the scaphoid of a 17-year-old female. GCTB typically occurs in the epiphysis of long bones, such as the distal femur, proximal tibia, and distal radius, with a higher incidence in patients 20-50 years of age. Most GCTBs of the hand are found in tubular bones, the metacarpals, and phalanges; Averill et al. reviewed 1,228 GCTs, of which 31 were found in the hand with only a single one located in the scaphoid [8]. The six previously reported cases of GCTB in the scaphoid were in adult patients older than ours: 18-year-old male, 19-year-old female, 26-year-old male, 29-year-old female, 30-year-old female, and 48-year-old female [14, 15, 21–24].

Usually, the giant-cell tumor of bone located in the hand is treated with intralesional curettage and burr and with bone grafting or bone cement; alternatively, it may be treated by wide excision with reconstruction. Amputation is considered in refractory cases or if the tumor involves an expendable bone. Studies have compared the rate of local recurrence associated with these procedures. In a retrospective review of 327 cases of GCTB in any bone, Campanacci et al. found local recurrence rates of 27% in patients treated with intralesional excision, 8% after marginal excision, and 0% after wide or radical resection [25]. Saikia et al. retrospectively reviewed the literature and showed a local recurrence rate of 75% (48/64) in the hand when patients were treated with intralesional procedures, compared to 24% (9/38) when treated with resection or ray amputation [26]. Similar findings were reported by Averill et al., who showed an 87% (13/15) recurrence rate of GCTB in the hand after curettage [8].

Management of patients with GCTB may also consist of denosumab and bisphosphonates. Denosumab, a monoclonal antibody against RANKL (receptor activator of nuclear factor kappa B [NF-*κ*B] ligand), prevents the development of osteoclasts thereby reducing bone resorption. Additionally, denosumab eliminates GCTB characteristic giant cells while concurrently building new bone [27, 28]. Through two phase-2 trials, denosumab has been predominantly recommended for patients with unresectable GCTB or when surgery might result in severe morbidity [29, 30]. Bisphosphonates have also been found to reduce the destruction caused by GCTB through inducing apoptosis [31]. Preoperative treatment with bisphosphonates has not shown the ability to eradicate the tumor but has shown a decrease in recurrence in stage III diseases [12, 32]. Although medical management seems to more dramatically decrease the osteoclastic nature of GCTB in advanced disease, further studies are needed to demonstrate the role and complications of bisphosphonates and denosumab in the long-term treatment of patients with less aggressive disease.

In patients presenting with a Campanacci grade 3 giant-cell tumor in a carpal bone, we believe that surgeons should consider a more aggressive primary treatment like excision. Although curettage with adjuvant therapy has demonstrated improved wrist function in other studies compared to excision and reconstruction, the recurrence rate is significantly higher which warrants a more definitive treatment. For GCTB of the scaphoid, more definitive treatment would be en bloc excision with a reconstruction, like four-corner fusion or proximal row carpectomy. Patients presenting with Campanacci grade 2 carpal lesions deserve an intraoperative assessment of bone quality and carpal mechanics after curettage has been performed to determine if the risk of recurrence outweighs the improved functional outcome that grafting, or cement, would yield. When treating a patient with a Campanacci grade 1 lesion, curettage with adjuvant treatment is strongly recommended to preserve wrist mechanics since recurrence rates are lower.
